# Elimination of Incessant Ventricular Tachycardia in Ischemic Cardiomyopathy with High-density Grid Technology

**DOI:** 10.19102/icrm.2021.120111S

**Published:** 2021-01-15

**Authors:** Chirag R. Barbhaiya, Kara Metcalf, M. Reed Bonvissuto, Michael Spinelli, Anthony Aizer, Douglas Holmes, Larry A. Chinitz

**Affiliations:** ^1^Leon H. Charney Division of Cardiology, New York University Langone Health, New York, NY, USA; ^2^Abbott Medical, Inc., Minneapolis, MN, USA

**Keywords:** Extracorporeal membrane oxygenation, high-density grid, ischemic cardiomyopathy, ventricular tachycardia storm

A 76-year-old man with a history of prior anterior-wall myocardial infarction, left ventricular ejection fraction of 25%, and primary-prevention implantable cardioverter-defibrillator placement presented with ventricular tachycardia storm and 17 ICD shocks. Extracorporeal membrane oxygenation (ECMO) was initiated due to incessant ventricular tachycardia (VT) and hemodynamic instability. The VT cycle length was 490 ms, with left bundle branch morphology in lead V1 and negative concordance throughout the precordium. He was brought in for urgent VT ablation and concurrent left ventricular, endocardial, high-density electroanatomic maps were created of the clinical arrhythmia and right ventricular pacing using the EnSite Precision™ cardiac mapping system and a multielectrode grid mapping catheter (Advisor™ HD Grid Mapping Catheter, Sensor Enabled™). A large low-voltage area of 95.7 cm^2^ was identified, within which the full diastolic pathway was visualized **(Figure 1 and Video 1)**. The clinical arrhythmia terminated following two seconds of radiofrequency application at a site with early diastolic activation and was not again observed again.

Following subsequent substrate modification guided by targeting of the crowding identified during sinus rhythm isochronal late-activation mapping and fractionation mapping, the patient was noninducible for any ventricular arrhythmia after programmed extrastimulation at two base cycle lengths and up to three extrastimuli. ECMO was decannulated on the second postoperative day and the patient was discharged on the fifth postoperative day. He remained free from recurrent arrhythmia at more than 45 days of follow-up.

The identification of critical locations for reentrant VTs related to large scars can be challenging and labor-intensive. In this case, high-density automated mapping using the Advisor™ HD Grid catheter facilitated rapid identification of the critical site of the clinical VT, while the ablation of areas of automatically identified ILAM crowding and fractionated electrograms rendered the patient noninducible for VT.

## Figures and Tables

**Figure 1:**
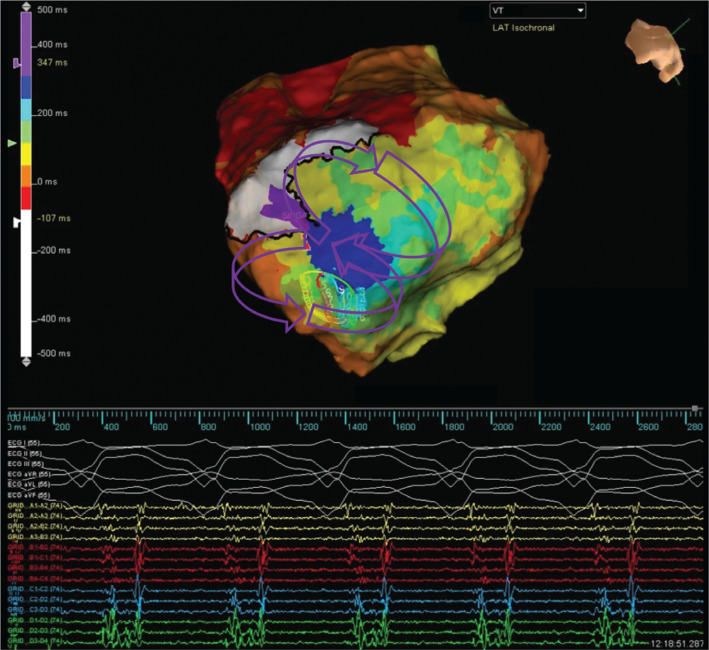
High-density activation map of the clinical VT.

**Video 1.** Rapid, high-density, multimodality mapping and catheter ablation of incessant ventricular tachycardia in ischemic cardiomyopathy.

